# Application of CT-Based Radiomics in Discriminating Pancreatic Cystadenomas From Pancreatic Neuroendocrine Tumors Using Machine Learning Methods

**DOI:** 10.3389/fonc.2021.606677

**Published:** 2021-07-22

**Authors:** Xuejiao Han, Jing Yang, Jingwen Luo, Pengan Chen, Zilong Zhang, Aqu Alu, Yinan Xiao, Xuelei Ma

**Affiliations:** ^1^ Department of Biotherapy, Cancer Center, West China Hospital, Sichuan University, Chengdu, China; ^2^ State Key Laboratory of Oncology in South China, Collaborative Innovation Center for Cancer Medicine, Sun Yat-sen University Cancer Center, Guangzhou, China; ^3^ Melanoma and Sarcoma Medical Oncology Unit, State Key Laboratory of Oncology in South China, Collaborative Innovation Center for Cancer Medicine, Sun Yat-sen University Cancer Center, Guangzhou, China; ^4^ West China School of Medicine, West China Hospital, Sichuan University, Chengdu, China

**Keywords:** pancreatic cystadenomas, pancreatic neuroendocrine tumors, radiomics, machine learning, differentiation, pNETs, CT

## Abstract

**Objectives:**

The purpose of this study aimed at investigating the reliability of radiomics features extracted from contrast-enhanced CT in differentiating pancreatic cystadenomas from pancreatic neuroendocrine tumors (PNETs) using machine-learning methods.

**Methods:**

In this study, a total number of 120 patients, including 66 pancreatic cystadenomas patients and 54 PNETs patients were enrolled. Forty-eight radiomic features were extracted from contrast-enhanced CT images using LIFEx software. Five feature selection methods were adopted to determine the appropriate features for classifiers. Then, nine machine learning classifiers were employed to build predictive models. The performance of the forty-five models was evaluated with area under the curve (AUC), accuracy, sensitivity, specificity, and F1 score in the testing group.

**Results:**

The predictive models exhibited reliable ability of differentiating pancreatic cystadenomas from PNETs when combined with suitable selection methods. A combination of DC as the selection method and RF as the classifier, as well as Xgboost+RF, demonstrated the best discriminative ability, with the highest AUC of 0.997 in the testing group.

**Conclusions:**

Radiomics-based machine learning methods might be a noninvasive tool to assist in differentiating pancreatic cystadenomas and PNETs.

## Introduction

Pancreatic neuroendocrine tumors (PNETs), a rare group of heterogeneous tumors originated from ductal pluripotent stem cells, account for less than 5% of pancreatic neoplasms and 7% of all NETs (1–3). Its incidence has increased in recent years, reaching 0.48 per 100 000 persons per year in the United States. This increase is probably due to the improvement in modern imaging and endoscopic technologies (4). Based on the clinical manifestations, PNETs have two subtypes, functional and non-functional PNETs. The most common functional PNETs includes insulinoma, gastrinoma, and glucagonoma. However, about 2/3 of all PNETs are non-functional, making the diagnosis difficult in clinical practice (5). For localized and advanced, but resectable PNETs, surgery is the first-line therapy capable of improving the clinical outcome (6–8). Pancreatic cystadenomas including serous pancreatic cystadenomas and mucinous pancreatic cystadenomas, account for approximately 46.3% of all surgically removed pancreatic cystic tumors (9). Serous cystadenomas are rare glycogen-rich lesions, which arise from pancreatic ductal epithelium (10). But mucinous cystadenomas are cystic epithelial tumors, consisting of ovarian stromata and mucus-producing columnar epithelium (11). In clinical practice, most pancreatic cystadenoma patients are asymptomatic or manifest non-specific symptoms (12). The management of patients is different due to the biological differences of PNETs and pancreatic cystadenomas. The endoscopic ultrasound fine-needle aspiration (EUS-FNA) is considered the best approach to diagnosis pancreatic tumors, but it is invasive and not completely accurate due to the small size of samples (13, 14). Therefore, a preoperative differential diagnosis is vital to identify the most appropriate therapies and improve clinical management.

Generally, computed tomography (CT) is the most effective imaging technique for initial detection and tumor staging among pancreatic patients (15–18). Previous studies showed that CT could clearly show the tumor site and boundary, maximum diameter, cyst wall characteristics, enhancement degree and other imaging signs, which may contribute to differentiating pancreatic cystadenoma from PNETs (18–21). Radiomics is a high-throughput method to extract quantitative imaging features, which can conduct mining and analysis of image feature data in depth. The strength of radiomics is to provide an objective, repeatable, non-invasive and low-risk diagnostic tool, which helps to derive a comprehensive characterization of tumors heterogeneity. It has plenty of applications including biological feature prediction and tumor classification (14, 22, 23). According to recent studies, the radiological features extracted from CT images are helpful for differentiating pancreatic neoplasms (19, 24, 25), as well as the prediction of PNETs grading (21). For example, He et al. developed three models to differentiate non-functional PNET and pancreatic ductal adenocarcinoma (PDAC), which all showed good performance (26). The AUC of the validation cohort was 0.884 in the LASSO based model that integrated clinicoradiological features and radiomic. Another study built a radiomics model based on non-contrast MRI to predict grades of non-functional PNETs and the AUC was 0.769 in the training cohort and 0.729 in the validation cohort (27). However, there are no studies to differentiate pancreatic cystadenomas from PNETs. Therefore, we conducted this study to identify pancreatic cystadenomas and PNETs using machine learning methods based on enhanced CT image features.

## Materials and Methods

### Patient Selection

**Figure 1:**
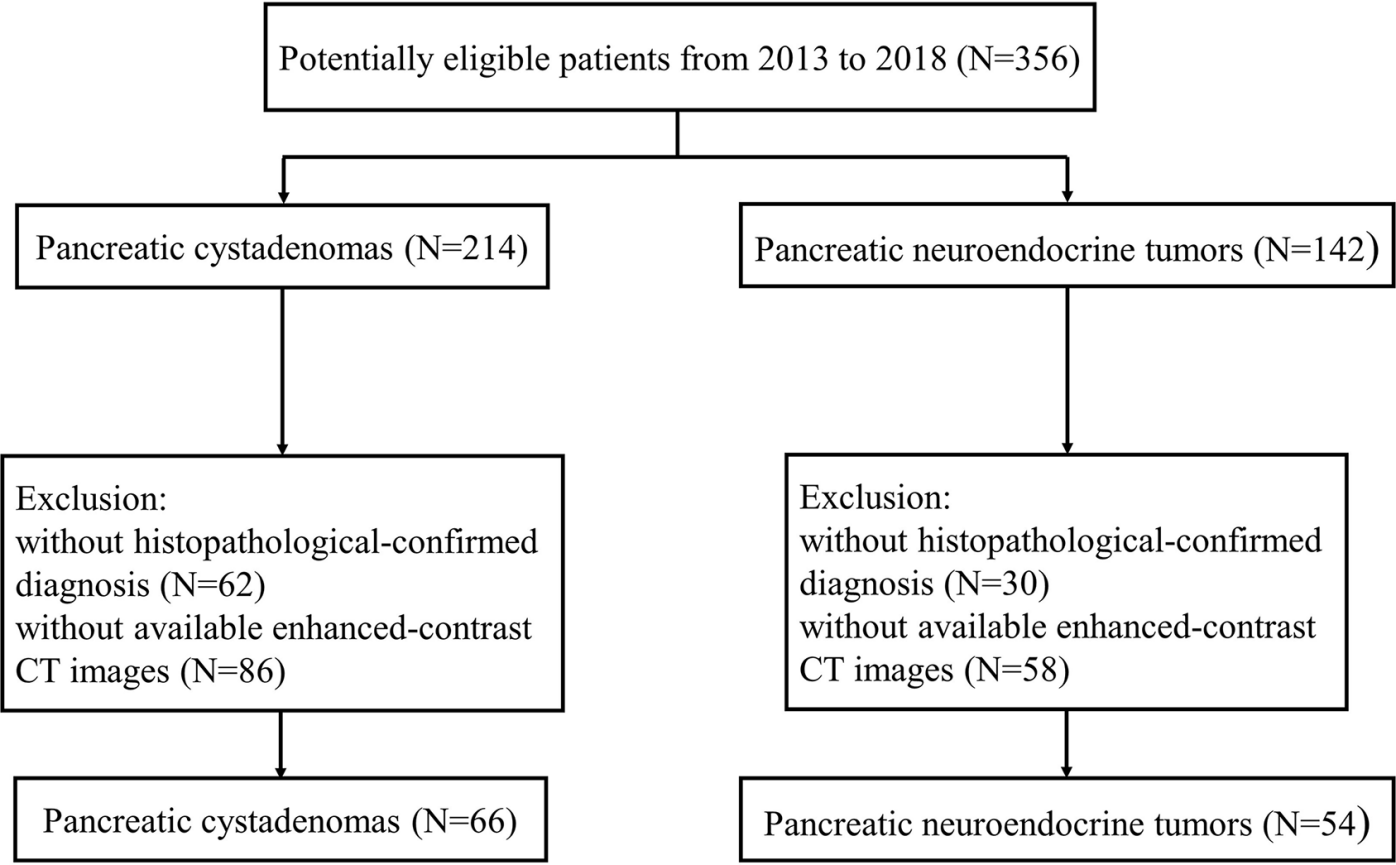
Flowchart shows selection of study population and exclusion criteria.

We retrospectively included all patient with pancreatic cystadenomas including pancreatic mucinous and serous cystadenoma or PNETs in West China hospital from January 2013 to May 2018 in this study. We initially identified 356 eligible patients according to criteria as followed: (1) pathological diagnosis of pancreatic cystadenomas or PNETs; (2) enhanced-contrast CT examination before biopsy or surgery. Then 92 of 356 patients without exact pathological evidence supporting pancreatic cystadenomas or PNETs were excluded. In addition, 144 patients lacking abdominal enhanced-contrast CT images before surgery were also excluded. Finally, we enrolled 120 qualified patients, consisting of 66 pancreatic cystadenomas patients and 54 PNETs patients. The patient selection process was illustrated in **Figure 1**. All procedures conformed to the Declaration of Helsinki, as well as its later amendments.

### CT Image Acquisition

All the contrast-enhanced CT images were collected from the patients before they received any treatment. A single 64-MDCT scanner (Brilliance64, Philips Medical Systems, Eindhoven, The Netherlands) were used for scanning. The tube voltage was 120 kVp, and tube current was 200 mAs. The gantry rotation time was 0.42 s, and the detector collimation was 0.75 mm. The matrix was 512×512 and beam pitch was 0.891. The slice thickness was 1.0 mm and reconstruction increment was 5.0 mm. Before the examination, patients were administrated with 1.5-2.0 mL/kg of nonionic contrast material (Omnipaque 350, GE Healthcare) intravenously at 3 mL/s. Images were obtained at 60-65 s for portal venous phase.

### ROI Segmentation and Texture Extraction

**Figure 2:**
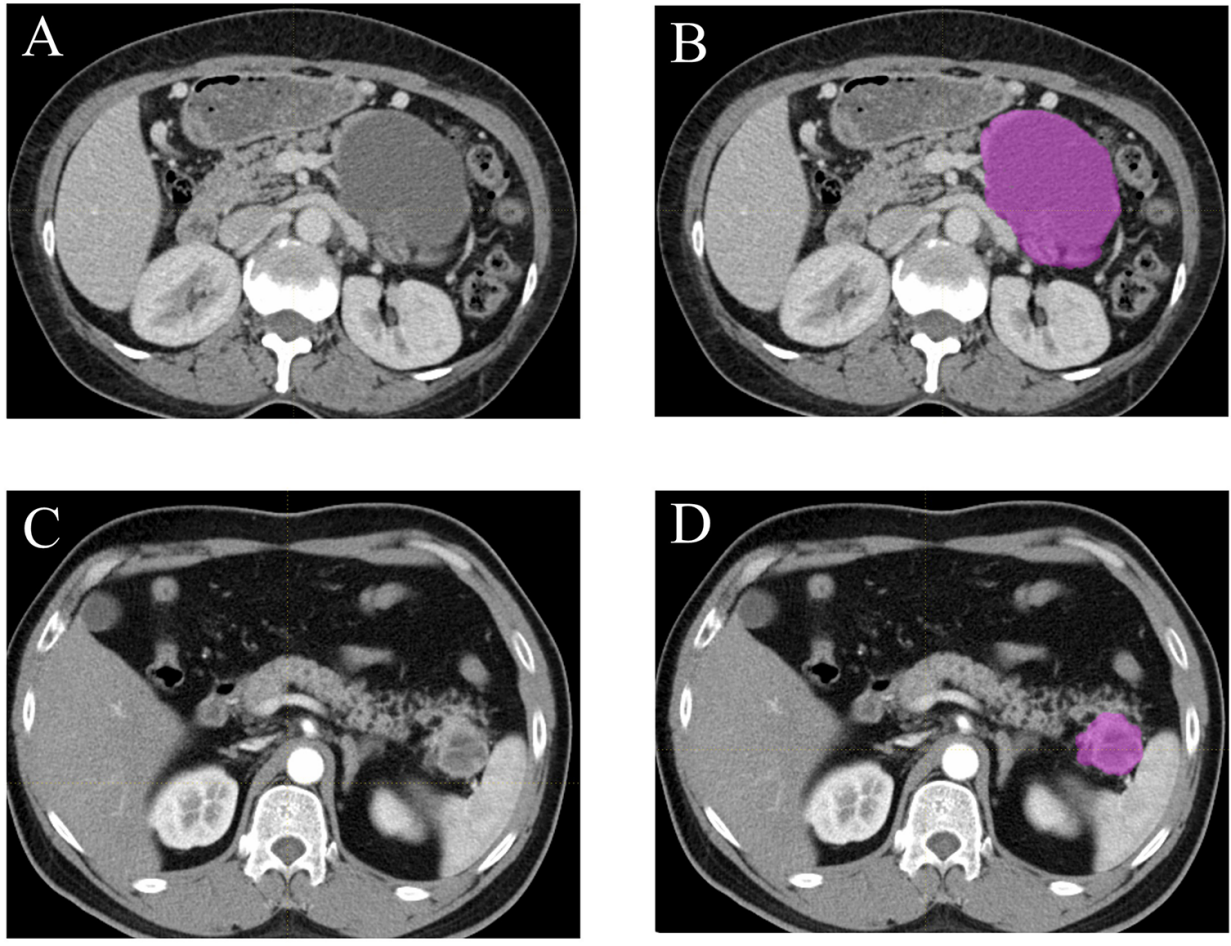
Examples of lesion segmentation and contouring on contrast-enhanced CT images. Portal vein phase CT images of a patient with histopathologically proved pancreatic cystadenomas **(A, B)** and pancreatic neuroendocrine tumors **(C, D)**.

All the contrast-enhanced CT images (400-bit gray scale) were obtained through searching the picture archiving and communication system (PACS). The lesion segmentation and texture analysis were acquired with a local image features extraction software (LIFEx v3.74, CEA-SHFJ, Orsay, France) (9). Briefly, the two-dimensional region of interest (ROI) was firstly obtained by drawing the outline of each tumor in the portal vein phase CT images (**Figure 2**) and then three-dimensional ROIs were automatically generated with default setting (28, 29). In order to reduce bias in the evaluation of derived radiomic features, the whole process was performed independently by two experienced radiologists without relevant knowledge of pancreatic tumor diagnosis and a third radiologist evaluated and selected the more accurate ROIs. The ROIs included the whole tumor while avoiding vascular shadows and surrounding adipose tissue. Then the textural parameters were calculated automatically based on the ROIs by the computer software LIFEx. Forty-eight texture features were extracted, including histogram-based matrix, shape-based matrix, gray-level co-occurrence matrix (GLCM), gray-level run length matrix (GLRLM), neighborhood gray-level dependence matrix (NGLDM) and gray-level zone length matrix (GLZLM). The association among texture parameters was analyzed by Pearson correlation coefficient test.

### Machine Learning Model

**Figure 3:**
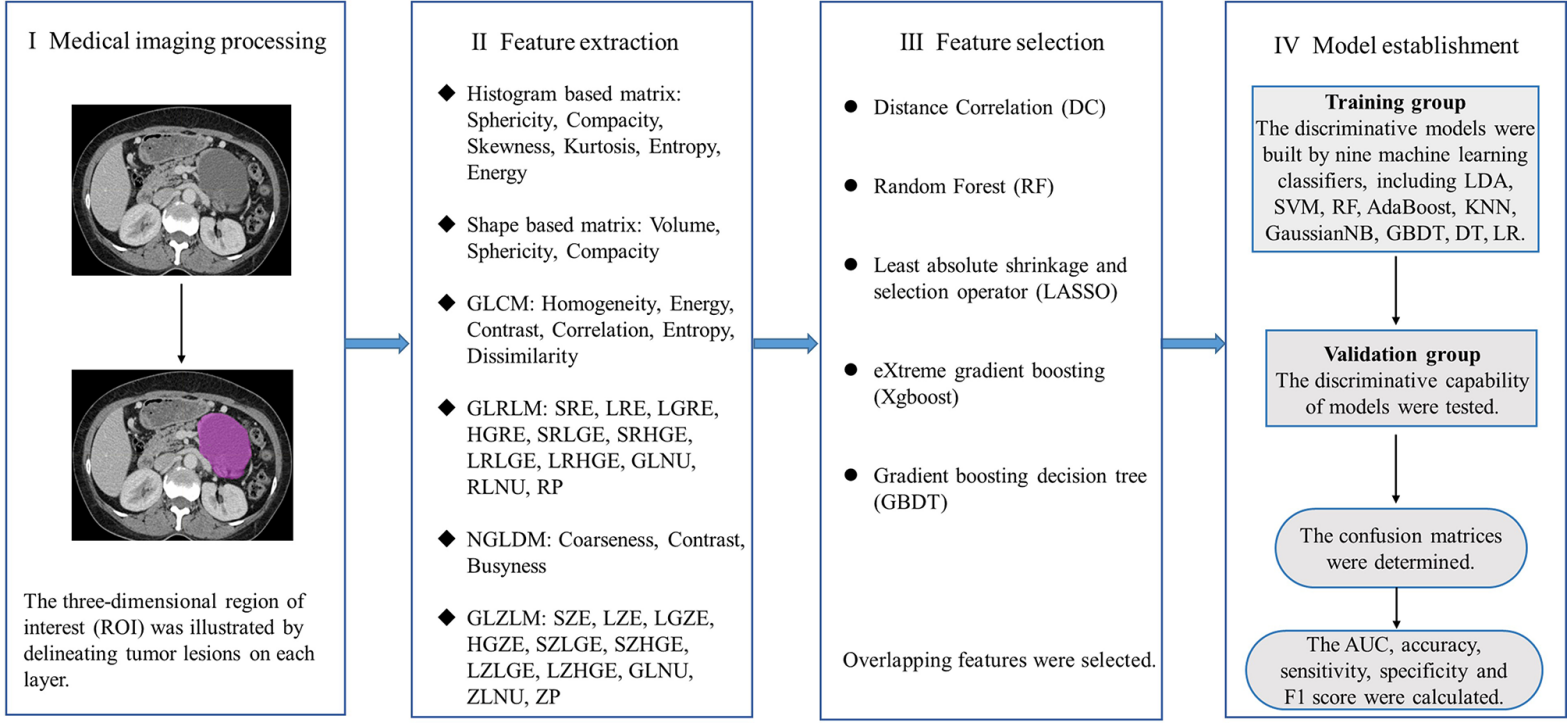
Flowchart depicts the workflow of the whole study.

In this study, five feature selection methods, namely, random forest (RF), distance correlation (DC), least absolute shrinkage and selection operator (LASSO), gradient boosting decision tree (GBDT) and eXtreme gradient boosting (Xgboost) were used to analyze the texture parameters and several clinicoradiological features (gender, age, size, location of lesions and calcification of lesions). Then, discriminative models were built by nine machine learning classifiers, which covers linear discriminant analysis (LDA), RF, Adaboost, support vector machine (SVM), GaussianNB, k-nearest neighbor (KNN), GBDT, logistic regression (LR) and decision tree (DT). Patients meeting the criteria were randomly assigned to either training group or validation group. The ration of patient number in training group to validation group was 4:1. The classification models were generated from the training group and the discriminative capability of models were tested in the validation group. We performed 10-fold cross validation with 1000 repetition to guarantee the real distribution of classification. The sensitivity, specificity, accuracy and F1 score were calculated accordingly after establishing a confusion matrix. Besides, the area of receiver operating characteristic curve (AUC) was recorded to assess the discriminative ability of the classification models. The machine learning algorithm was completed by the Python programming language and sklear-Package. The whole study process was shown in **Figure 3**.

## Results

### Patient Characteristics

In this study, a total number of 120 patients (including 66 individuals with pancreatic cystadenomas and 54 patients with PNETs) were enrolled. The median age of all patients was 50.26 (ranging from 24 to 77) in the group of pancreatic cystadenomas, and 54 (ranging from 29 to 73) in the PNETs group. Of the 66 patients with pancreatic cystadenomas, 15 (22.7%) were males and 51 (77.3%) were females. In PNETs group, there were 32 (59.3%) male and 22 (40.7%) female patients. In the patients with pancreatic cystadenomas, 40.9% of lesions were located in the pancreatic head, whereas 59.1% were in the pancreatic body-tail. Of the 54 patients with PNETs, the tumor was located in the head of pancreas in 23 patients (42.6%) and in the pancreatic body-tail in 31 patients. The average size of pancreatic cystadenomas and PNETs was 4.1 cm (range 0.8-12.0 cm) and 4.19 cm (range 1.0-12.0 cm) respectively. The summary of patient and lesion characteristics were recorded in **Table 1**.

**Table 1:** Characteristics of patients and lesions.

Characteristics	Pancreatic cystadenomas	Pancreatic neuroendocrine tumors
**Number**	66	54
**Mean age (range) (year)**	50.26 (24-77)	50.48 (29-73)
**Gender**		
Male	15 (22.7%)	32 (59.3%)
Female	51 (77.3%)	22 (40.7%)
**Location**		
Head	27 (40.9%)	23 (42.6%)
Body or tail	39 (59.1%)	31 (57.4%)
**Maximum diameter (range) (cm)**	4.1 (0.8-12)	4.19 (1-12)

### Texture Parameters Selection

**Figure 4:**
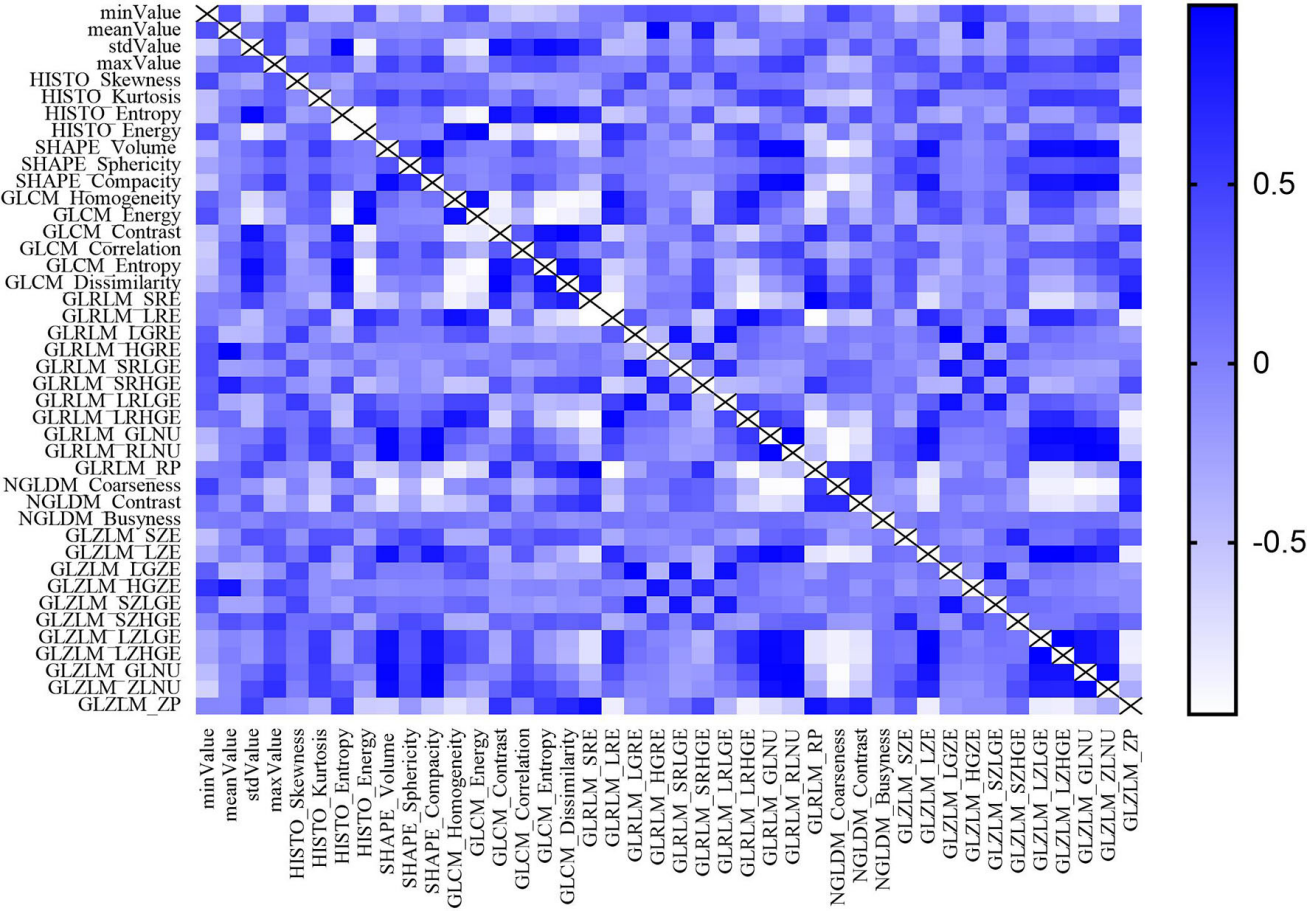
The result of Pearson correlation coefficients test between radiomics features.

Most texture parameters were uncorrelated or weakly correlated according to the result of Pearson correlation coefficient test, only a few parameters showing strong positive or negative correlation (**Figure 4**). Different texture parameters were selected in five feature selection methods, however, no clinicoradiological feature was selected using the five feature selection methods (**Table 2**). LASSO selected the most number of parameters, including maxValue, HISTO_Skewness, SHAPE_Sphericity, GLRLM_SRLGE, GLRLM_GLNU, GLRLM_RLNU, GLRLM_RP, GLZLM_SZE, GLZLM_LGZE, GLZLM_SZLGE, GLZLM_LZLGE, GLZLM_GLNU, GLZLM_ZLNU, GLZLM_ZP and Xgboost selected only four parameters.

**Table 2:** Selected features of five selection methods.

DC	RF	LASSO	Xgboost	GBDT
meanValue	meanValue	maxValue	meanValue	HISTO_Kurtosis
HISTO_Kurtosis	HISTO_Kurtosis	HISTO_Skewness	HISTO_Kurtosis	GLRLM_SRHGE
GLZLM_HGZE	PARAMS_YSpatialResampling	SHAPE_Sphericity	GLRLM_HGRE	GLRLM_LRHGE
GLZLM_SZHGE	GLRLM_HGRE	GLRLM_SRLGE	GLRLM_LRHGE	GLZLM_HGZE
GLRLM_HGRE	GLRLM_SRHGE	GLRLM_GLNU		GLZLM_SZHGE
GLRLM_SRHGE	GLRLM_LRHGE	GLRLM_RLNU		
GLZLM_SZE	GLZLM_SZE	GLRLM_RP		
	GLZLM_HGZE	GLZLM_SZE		
	GLZLM_SZHGE	GLZLM_LGZE		
		GLZLM_SZLGE		
		GLZLM_LZLGE		
		GLZLM_GLNU		
		GLZLM_ZLNU		
		GLZLM_ZP		

DC, distance correlation; RF, random forest; LASSO, least absolute shrinkage and selection operator; Xgboost, eXtreme gradient boosting; GBDT, gradient boosting decision tree; GLZLM, gray-level zone length matrix; HGZE, High Grey Level Zone Emphasis; GLRLM, Gray Level Run Length Matrix; SZHGE, Short Zone High Grey Level Emphasis; HGRE, High Gray Level Run Emphasis; SRHGE, Short-Run High Grey Level Emphasis; SZE, Short Zone Emphasis; LRHGE, Long-Run High Grey Level Emphasis; SRLGE, Short-Run Low Grey Level Emphasis; GLNU, Grey Level Non-Uniformity; RLNU, Run Length Non-Uniformity; RP, Run Percentage; LGZE, Low Gray Level Zone Emphasis; SZLGE, Short Zone Low Grey Level Emphasis; LZLGE, Long Zone Low Grey Level Emphasis; ZP, Zone Percentage.

### Machine-Learning Model Evaluation

**Figure 5:**
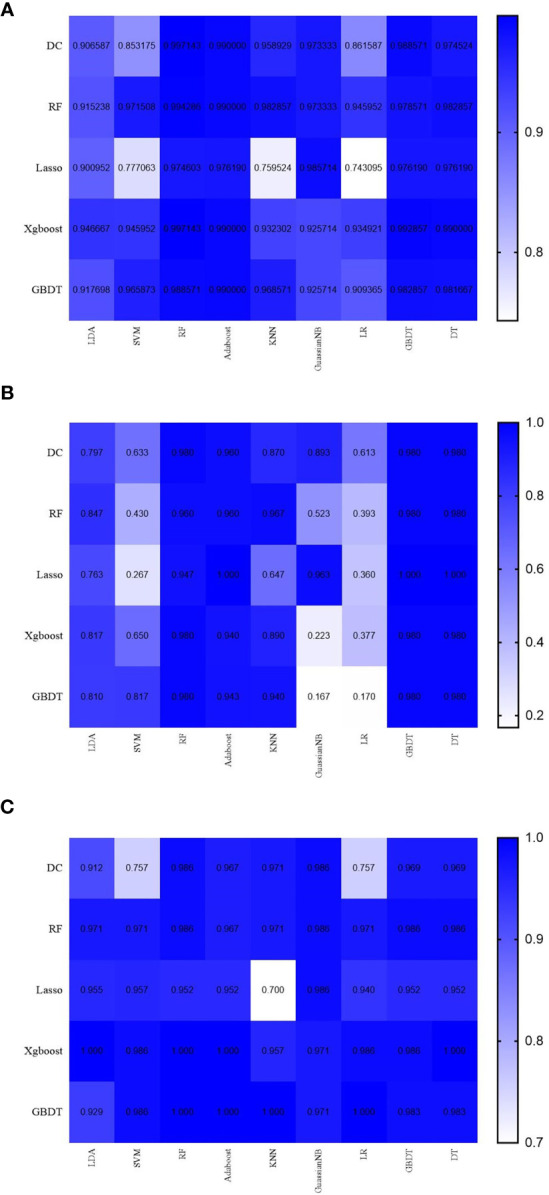
The results of AUC **(A)**, sensitivity **(B)** and specificity **(C)** in the testing group.

**Figure 6:**
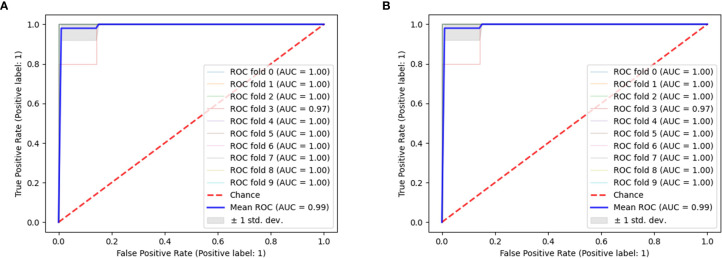
The ROC curves of 10 fold for DC+RF **(A)** and Xgboost+RF **(B)** in the testing group.

Through a random combination of five feature selection methods and nine machine-learning classifiers, we acquired a total of forty-five predictive models for distinguishing pancreatic cystadenomas from PNETs. The AUC, accuracy, sensitivity, specificity and F1 score of all models in the testing group were shown in **Table 3**. The result revealed that radiomics-based machine learning models were able to differentiate pancreatic cystadenoma from PNETs, with AUC more than 0.743 in the validation cohort (**Figure 5**). It is noteworthy that the combination of DC as the selection method and RF as the classifier, as well as Xgboost+RF, demonstrated the best discriminative ability, with the highest AUC of 0.997 in the testing group. The receiver operating characteristic (ROC) curves of 10 fold for DC+RF and Xgboost+RF in the testing group were shown in **Figure 6**. For the model (DC+RF) in the testing group, the accuracy, sensitivity, specificity and F1 score were 0.983, 0.980, 0.986 and 0.980, respectively. The mean AUC for DC+RF was 0.9977 (Std= 0.0024; 95% CI, 0.9976 to 0.9979) after 1000 repetition. As for the model of Xgboost+RF, the accuracy, sensitivity, specificity and F1 score were 0.992, 0.980, 1.000 and 0.989 respectively in the validation group and the mean AUC was 0.9962 (Std=0.0033; 95% CI, 0.9960 to 0.9964) after 1000 repetition.

**Table 3:** Results of discriminative models in distinguishing pancreatic cystadenomas and pancreatic neuroendocrine tumors in the testing group.

	DC					RF					LASSO					Xgboost					GBDT				
	AUC	Accuracy	Sensitivity	Specificity	F1-score	AUC	Accuracy	Sensitivity	Specificity	F1-score	AUC	Accuracy	Sensitivity	Specificity	F1-score	AUC	Accuracy	Sensitivity	Specificity	F1-score	AUC	Accuracy	Sensitivity	Specificity	F1-score
**LDA**	0.907	0.858	0.797	0.912	0.832	0.915	0.917	0.847	0.971	0.890	0.901	0.867	0.763	0.955	0.832	0.947	0.917	0.817	1.000	0.894	0.918	0.875	0.810	0.929	0.850
**SVM**	0.853	0.700	0.633	0.757	0.653	0.972	0.725	0.430	0.971	0.542	0.777	0.642	0.267	0.957	0.365	0.946	0.833	0.650	0.986	0.764	0.966	0.908	0.817	0.986	0.886
**RF**	0.997	0.983	0.980	0.986	0.980	0.994	0.975	0.960	0.986	0.969	0.975	0.950	0.947	0.952	0.948	0.997	0.992	0.980	1.000	0.989	0.989	0.992	0.980	1.000	0.989
**Adaboost**	0.990	0.967	0.960	0.967	0.961	0.990	0.967	0.960	0.967	0.961	0.976	0.975	1.000	0.952	0.977	0.990	0.975	0.940	1.000	0.964	0.990	0.975	0.943	1.000	0.966
**KNN**	0.959	0.925	0.870	0.971	0.908	0.983	0.967	0.967	0.971	0.964	0.760	0.675	0.647	0.700	0.634	0.932	0.925	0.890	0.957	0.912	0.969	0.975	0.940	1.000	0.967
**GaussianNB**	0.973	0.942	0.893	0.986	0.928	0.973	0.775	0.523	0.986	0.654	0.986	0.975	0.963	0.986	0.971	0.926	0.633	0.223	0.971	0.292	0.926	0.608	0.167	0.971	0.209
**LR**	0.862	0.692	0.613	0.757	0.633	0.946	0.708	0.393	0.971	0.492	0.743	0.675	0.360	0.940	0.458	0.935	0.708	0.377	0.986	0.502	0.909	0.625	0.170	1.000	0.276
**GBDT**	0.989	0.975	0.980	0.969	0.972	0.979	0.983	0.980	0.986	0.980	0.976	0.975	1.000	0.952	0.977	0.993	0.983	0.980	0.986	0.980	0.983	0.983	0.980	0.983	0.981
**DT**	0.975	0.975	0.980	0.969	0.972	0.983	0.983	0.980	0.986	0.980	0.976	0.975	1.000	0.952	0.977	0.990	0.992	0.980	1.000	0.989	0.982	0.983	0.980	0.983	0.981

DC, distance correlation; RF, random forest; LASSO, least absolute shrinkage and selection operator; Xgboost, eXtreme gradient boosting; GBDT, gradient boosting decision tree; LDA, linear discriminant analysis; SVM, support vector machine; KNN, k-nearest neighbor; LR, logistic regression; DT, decision tree; AUC, area under curve.

## Discussion

Contrast‐enhanced CT and MRI are widely used for identification of pancreatic neoplasms, which is not sufficiently specific due to the overlapping of anatomical imaging features between different pancreatic tumors (30). Furthermore, PNETs identification is to exploit somatostatin analog radiotracers for CT and MRI imaging, which is easily affected by intra-/inter-observer variability (31). The accuracy for manual identification of tumor lesions is only 60%-70%, even by highly-trained radiologists (32). To date, EUS-FNA is considered the best approach to diagnosis pancreatic tumors based on cytopathologic features. However, it is invasive with the possibility for puncture failure, as well as unable to reflect tumor heterogeneity accurately due to the small size and limited number of samples (14, 33).

Radiomics combine quantitative image analysis with machine learning or artificial intelligence methods to select out and classify target features in the sample images. It has been applied in modern medical care including diagnosis, risk stratification, virtual biopsy and radiogenomics (34). Several studies have investigated the utility of machine learning based-radiomics on the differentiation of pancreatic mucinous cystadenomas from pancreatic serous cystadenomas (35, 36) and the prediction of PNETs grading (13, 21, 37–39). However, no studies reported how to differentiate pancreatic cystadenomas from PNETs. Given the similar incidence, nonspecific symptoms and various biological behaviors of pancreatic cystadenomas and PNETs, we conducted this study by combining radiomics and machine learning method to distinguish these two types of pancreatic lesions. This is the first study that utilized enhanced CT images features and machine learning methods to differentiate pancreatic cystadenomas from PNETs so far.

Different radiomic features were obtained from preoperative contrast-enhanced CT images. Then the establishment of predictive models were based on five feature-selection methods and nine machine-learning classifiers. Our results proved that the combination of predictive models and appropriate selection methods could differentiate pancreatic cystadenomas from PNETs, with AUC more than 0.743 in the validation cohort. Notably, the combination of DC as the selection method and RF as the classifier, as well as Xgboost+RF, seemed to be optimal in differentiating the two types of pancreatic lesions, with the highest AUC of 0.997 in the testing group. For the model (DC+RF) in the testing group, the accuracy, sensitivity, specificity and F1 score were 0.983, 0.980, 0.986 and 0.980, respectively. In addition, the accuracy, sensitivity, specificity and F1 score were 0.992, 0.980, 1.000 and 0.989 respectively in the validation group for the model of Xgboost+RF. RF classifier is an excellent classification algorithm that has been widely used in many studies (40–42). Previous studies have investigated the diagnostic performance of PNETs from PDACs based on CT features and texture analysis. Yu et al. used LASSO and univariate logistic regression (ULR) analyses to select CT radiomic features and generated four multivariate logistic regression (MLR) models. The highest AUC was 0.926 in the model built with CT radiomic features extracted from portal venous phase (43). Another study developed a MLR model to discriminate PNETs from solid pseudopapillary tumors (SPTs) (44). The model incorporating MRI radiomics features and sex and age of patients exhibited the best discriminative ability with AUC of 0.97 and 0.86 in the training and validation cohort separately. Compared with previous studies, we developed more predictive models by employing more selection methods and classifiers to identify the best model.

However, our study existed several limitations. First of all, this study was conducted in a retrospective fashion, which had unavoidable selection bias. Second, it was a single-center study with a small group of patients. Multicenter studies with more patients would be helpful to confirm these findings. Third, there was subjectivity when manually defining the tumor boundary on CT images.

## Conclusion

This study concluded that radiomics-based machine learning method was able to preoperatively differentiate pancreatic cystadenomas from PNETs, which may guide clinical decision-making for better treatment. The feature selection method DC or Xgboost and classifier RF showed a good prospect in discriminating pancreatic cystadenomas from PNETs. However, more large-scale multicenter studies are required to supplement more evidence concerning the feasibility of this method in clinical practice.

## Data Availability Statement

The dataset generated for this study can be obtained from the correspondence author.

## Ethics Statement

The studies involving human participants were reviewed and approved by The Ethics Administration Office of the West China Hospital, Sichuan University. Written informed consent to participate in this study was provided by the participants’ legal guardian/next of kin.

## Author Contributions

XH and JY collected images, extracted features, performed statistical analysis and drafted the manuscript. JL collected image data and extracted texture feature. PC and ZZ collected image data and drafted the manuscript. AA and YX revised the manuscript. XM participated in study conception and manuscript revision. All authors contributed to the article and approved the submitted version.

## Conflict of Interest

The authors declare that the research was conducted in the absence of any commercial or financial relationships that could be construed as a potential conflict of interest.
